# Do national health insurance schemes guarantee financial risk protection in the drive towards Universal Health Coverage in West Africa? A systematic review of observational studies

**DOI:** 10.1371/journal.pgph.0001286

**Published:** 2023-08-09

**Authors:** Sydney N. N. T. Odonkor, Ferdinand Koranteng, Martin Appiah-Danquah, Lorena Dini

**Affiliations:** 1 Institute of Tropical Medicine and International Health, Charité Universitätsmedizin Berlin, Berlin, Germany; 2 Institute of General Practice and Family Medicine, Charité Universitätsmedizin Berlin, Berlin, Germany; 3 Life Sciences Institute, University of British Columbia, Vancouver, Canada; 4 Department of Surgery, NES Healthcare, Parkside Hospital, London, Wimbledon, United Kingdom; University of Ghana, GHANA

## Abstract

To facilitate the drive towards Universal Health Coverage (UHC) several countries in West Africa have adopted National Health Insurance (NHI) schemes to finance health services. However, safeguarding insured populations against catastrophic health expenditure (CHE) and impoverishment due to health spending still remains a challenge. This study aims to describe the extent of financial risk protection among households enrolled under NHI schemes in West Africa and summarize potential learnings. We conducted a systematic review following the PRISMA guidelines. We searched for observational studies published in English between 2005 and 2022 on the following databases: PubMed/Medline, Web of Science, CINAHL, Embase and Google Scholar. We assessed the study quality using the Joanna Briggs Institute (JBI) critical appraisal checklist. Two independent reviewers assessed the studies for inclusion, extracted data and conducted quality assessment. We presented our findings as thematic synthesis for qualitative data and Synthesis Without Meta-analysis (SWiM) for quantitative data. We published the study protocol in PROSPERO with ID CRD42022338574. Nine articles were eligible for inclusion, comprising eight cross-sectional studies and one retrospective cohort study published between 2011 and 2021 in Ghana (n = 8) and Nigeria (n = 1). While two-thirds of the studies reported a positive (protective) effect of NHI enrollment on CHE at different thresholds, almost all of the studies (n = 8) reported some proportion of insured households still encountered CHE with one-third reporting more than 50% incurring CHE. Although insured households seemed better protected against CHE and impoverishment compared to uninsured households, gaps in the current NHI design contributed to financial burden among insured populations. To enhance financial risk protection among insured households and advance the drive towards UHC, West African governments should consider investing more in NHI research, implementing nationwide compulsory NHI programmes and establishing multinational subregional collaborations to co-design sustainable context-specific NHI systems based on solidarity, equity and fair financial contribution.

## Introduction

According to the World Health Organization (WHO) in 2022, despite the fact that 75% of national health policies of countries worldwide are directed towards achieving UHC, half of the world’s population still lack access to the healthcare they need and more than 930 million people worldwide spend at least 10% of their household income on healthcare with approximately 100 million people worldwide impoverished each year due to out-of-pocket (OOP) health expenditure [1]. To attain UHC, countries must strive to achieve better progress on the three main dimensions of UHC which include maximizing the proportions of the population covered, the costs covered and the services included in the benefit packages, all of which could be supported by increasing pooled funds and thus ensuring financial risk protection when accessing healthcare [2, 3].

Financial risk protection has the goal of safeguarding people against catastrophic health expenditure (CHE) and impoverishment due to OOP payment for health services. It forms an integral component of UHC as it ensures that people get access to the essential healthcare services they need without being at risk of financial hardship due to health spending. Furthermore, financial risk protection serves as an interface between a country’s health system and other social determinants of wellbeing [4]. This is evidenced by the fact that, the choice to access health services will be made by an individual if it does not come at the cost of sacrificing other basic needs or imperative social circumstances such as food security, shelter and basic education due to the risk of financial hardship [4, 5]. The devastating effects of OOP health expenditure on financial risk protection have been explored by many economists and researchers in different countries as shown by studies published in Malawi, India, Chile and Spain reporting on CHE and impoverishment [6–9].

The introduction of health insurance schemes in countries thus seeks to avert the financial burden placed on households due to OOP payments for health services [4]. Health insurance is a contract that binds two parties where a policyholder (insured) contributes premiums to a third party payer or a government program (insurer) [10]. Health insurance schemes guarantee that funds collected are pooled to ensure efficient risk sharing among the insured in such a way that the cross-subsidies are from healthy to the sick, rich to the poor, young to the old and those without children towards those with children while eliminating the risk of any single insured member incurring impoverishment as a result of health expenditures [10–13]. In fact, studies conducted in Ethiopia, China and Vietnam have affirmed the protective effect of health insurance schemes against CHE and impoverishment due to health expenditure [14–16].

Studies have reported different systems through which modern public health insurance schemes are operationalized in countries, these include three main schemes: the National Health Insurance (NHI), the Social Health Insurance (SHI) and the Community-based health insurance (CBHI) [17, 18]. The NHI is a compulsory government-run insurance system whereby the government serves as the single third party payer and the policyholder is allowed to individually purchase a health insurance after which funds of the entire population are pooled at a national level [19]. Under the SHI funds for health financing are raised through compulsory premiums deducted directly from the employee’s payroll taxes. Funds are usually pooled and managed by more than one third party payer (mostly public or quasi-public organization) [17, 20]. The CBHI employs the concept of pooling funds for health financing, however funds are normally collected and pooled in a subgroup of the population, mostly at a community level [17, 21].

The health financing mechanisms in West Africa have been known to face many challenges such as general insufficient national income, dedication of majority of general government expenditure to non-health-related sectors, insufficient external revenue for health, high informal sector workforce resulting in limited government tax revenue for health and marked corruption in the public sector [22–24]. The low prioritization of the healthcare sector by governments of the West African subregion continues to undermine the objectives of UHC [22]. The majority of the population end up paying OOP for healthcare services which predisposes them to impoverishment [25].

In the early to mid-2000s, the health insurance system was introduced into West Africa to pool risk in order to address the issue of frequent OOP payments while improving equitable access to healthcare [26, 27]. The NHI scheme is the predominant scheme in most parts of West Africa because other schemes, especially the SHI schemes, are considered less feasible and sustainable in most parts of West Africa because the majority of the population active in the informal sector and have no fixed salaries thus making compulsory deductions from payroll taxes a major challenge [20].

Furthermore, studies report that the poor policy implementation, the administrative burden of premium collection, the inefficient pooling of health insurance funds as well as the inequitable allocation of resources have weakened the health insurance systems and placed an additional burden on the already challenged health financing mechanisms of West African countries [4, 28–30]. In order to improve on the existing NHI systems and facilitate the attainment of UHC in West Africa, it is important to examine the extent to which the current NHI systems contribute to the financial risk protection of insured households in West Africa.

In this systematic review, our research question was; are households in any West African country enrolled under NHI schemes at lower risk of CHE and impoverishment due to health spending as compared to uninsured households? Our main aim was to describe the extent of financial risk protection among households enrolled under NHI schemes in West Africa from published literature and our specific objectives were; 1. To ascertain the effect of NHI on CHE and impoverishment due health spending in West Africa. 2. To ascertain the proportion of households under NHI schemes incurring CHE and impoverishment due health spending in West Africa. 3. To describe the current NHI schemes and their financing mechanisms in West African countries. 4. To summarize existing recommendations on the development of NHI from published literature and formulate potential strategies to improve the extent of financial risk protection among those currently insured under NHI schemes in West Africa.

## Methods

### Study design and study registration

Our study was a systematic review conducted and reported following the Preferred Reporting Items for Systematic Reviews and Meta-Analyses (PRISMA) standards. We registered the review protocol with the International Prospective Register of Systematic Reviews (PROSPERO) with ID CRD42022338574 on 28th June 2022.

### Search strategy

After specifying the research question with the PECO (Population, Exposure, Comparison, Outcome) framework (Table 1) and conducting an initial literature review, we developed the final search strategy using keywords, synonyms and Medical Subject Headings (MeSH) terms related to NHI, financial risk protection and UHC in all West African countries in order to increase yield of articles found.

**Table 1:** Framework to structure the research question (PECO—Population, Exposure, Comparison, Outcome).

Framework item	Details
**Population**	Households in any West African country
**Exposure**	Insured households under NHI schemes
**Comparison**	Uninsured households
**Outcome**	CHE and impoverishment due to health spending

We searched four major electronic databases namely PubMed/Medline, Web of Science and Cumulative Index to Nursing and Allied Health Literature (CINAHL) via EBSCOhost and Embase via Ovid. We also manually searched for grey literature on Google Scholar using advanced search string to identify relevant articles that were not published. We run the last search of the databases on 1st May 2022.

After titles and abstracts screening, we applied citation tracking and snowballing methods on articles eligible for full-text screening to help find more related relevant articles that met the eligibility criteria. The complete search strategies for each electronic database are presented in supporting information (S1 Table).

### Eligibility criteria and study selection process

After screening titles and abstracts of studies, we assessed the eligibility of relevant full texts articles by applying various eligibility criteria. We considered studies eligible for inclusion if studies with available full text were:

- observational studies (cohort studies, cross-sectional studies and case-control studies) conducted in any West African country.
- written in English and published between 1st January 2005 and 1st May 2022. This year limitation was applied because the NHI was introduced into West Africa in the early to mid-2000s and as such published articles from 2005 to date yielded more relevant studies to answer the research question [26, 27].
- describing the NHI system in any West African country in relation to revenue raising, pooling of funds and purchasing of services.
- mentioning financial risk protection and/or UHC among populations enrolled into NHI schemes in any West African country.
- assessing CHE and/or impoverishment due to health spending among populations enrolled into NHI schemes in any West African country.
- estimating CHE using either total household expenditure/income or non-subsistence expenditure/income.

We excluded studies from the review if they:

- were reviews, protocol papers, letters, editorials, conference abstracts and poster presentations.
- had no clear aims and objectives or did not report on ethical approval.
- had households with private health insurance as a comparison group.

For the study selection, we downloaded the citation of studies identified through the electronic databases including abstracts and full texts and imported them into the Covidence online tool. The Covidence tool automatically removed all duplicates which two independent reviewers (SO and FK) confirmed. Both reviewers then screened the titles and abstracts and assessed the full-text by carefully applying the eligibility criteria. The reviewers discussed disagreements encountered in the process of title, abstract and full-text screening between and resolved them by consensus before moving to the next phase. The study selection process was presented in a PRISMA 2020 flowchart.

### Data extraction

We extracted data using a purposefully designed data extraction form. We pilot-tested the initial data extraction form using two randomly selected articles of the final included articles and subsequently revised the form to ensure that all relevant information was captured by the final data extraction form.

Information extracted using the final data extraction form included the publication details (authors, year, title and citation), the study and participant characteristics and the exposure characteristics. We also extracted the primary and secondary outcome measures, the effect measures as well as additional information from authors of the articles. The exposure of interest was insurance status as defined by household enrolment into NHI schemes or not. We recorded the criteria for classifying household as insured as well as the total number of insured households in the sample population, the health insurance type and information on its financing mechanism. We also collected information about confounding variables.

We recorded the outcome parameters which included the mean household income/expenditure, the measure of financial risk protection as well as the respective thresholds used for the measure of CHE and poverty in the selected articles. We also captured the various effect measures used for assessing the association between the exposure and primary outcome. Furthermore, we recorded the proportion of CHE and impoverishment at the various thresholds as reported in the selected articles. For secondary outcomes, we considered any information on determinants of CHE and impoverishment as well as health outcomes and service utilization among insured populations so long as they were reported in the articles. Finally, we extracted additional information such as limitations, recommendations, study funding sources, concerns about bias and possible conflicts of interest.

Two reviewers (SO and MAD) independently extracted data from each study using the final data extraction form. The primary reviewer (SO) then examined all the extracted data for consistency and accuracy. The two reviewers discussed and resolved by consensus all discrepancies in the extracted data. The complete data extracted onto the data extraction form is presented in S1 Data.

### Quality appraisal

The final included studies were cross-sectional and cohort studies. To assess their quality, we applied the corresponding checklists of the Joanna Briggs Institute (JBI) Critical Appraisal Checklists [31–33]. Two reviewers (SO and FK) independently carried out the assessment and settled discrepancies by discussions until consensus was reached. We computed the inter-rater reliability (Cohen’s kappa score) and the percentage of agreement between the two reviewers using the Idostatistics online statistical tool [34]. We set a scoring system with three categories (high, moderate and low) to allow for comparison of the quality of studies in a transparent manner. For cross-sectional studies, we considered a score of four or less as low quality, a score of five and six as moderate quality and a score of seven and eight as high quality. For cohort studies, we considered a score of six or less as low quality, a score of seven to nine as moderate quality and a score of ten or more as high quality.

### Data synthesis and analysis

We performed a narrative synthesis which included an iterative process that consisted of two methods: the thematic synthesis for the qualitative data and the Synthesis Without Meta-analysis (SWiM) which provided a more transparent approach for the synthesis of quantitative data [35]. We conducted the thematic synthesis by assigning codes generated from identifying recurring themes in the data. Furthermore, we used the NVivo Release 1.6.2 software to organize, sort, arrange and summarize qualitative data. For the SWiM method, we used vote counting to capture the direction of effect of NHI on CHE and impoverishment as reported by the included studies. For the association between the exposure and the outcome for each study, we considered three possible effects which included, a positive effect represented by “+”, a negative effect represented by “−” and no statistically significant effect (p-value >0.05) represented by “0”. There was heterogeneity in the measurement of exposure (different criteria for classifying households as insured), measurement of outcomes (varying thresholds for CHE and impoverishment) as well as in the measurement of effect measures among the final included studies. These methodological and statistical heterogeneity did not allow for a formal meta-analysis. The collated elements of the narrative synthesis are presented in tables and texts under topics relevant to the objectives of the study (see Results).

## Results

### Study selection

**Fig 1:**
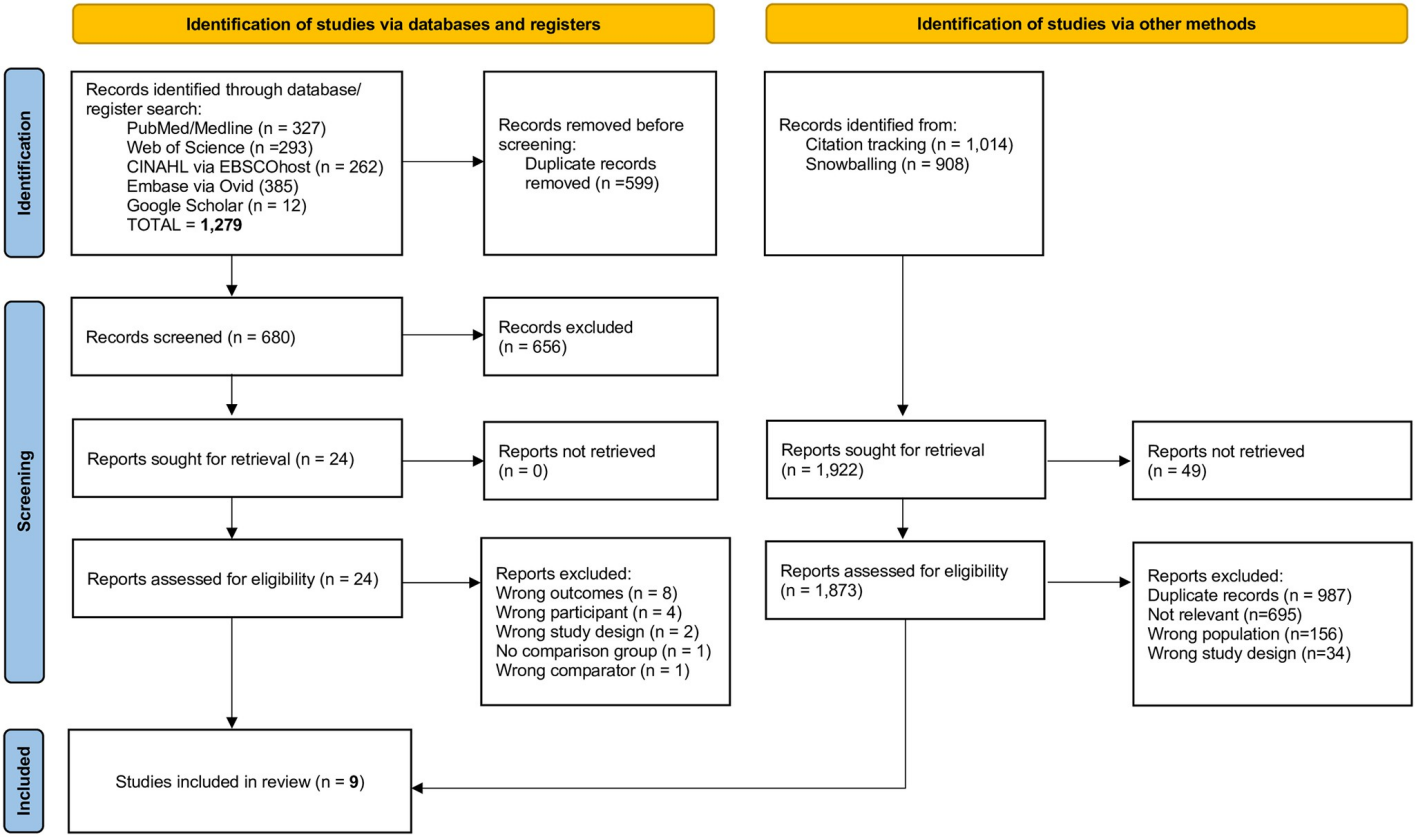
PRISMA 2020 flowchart showing the study selection process. Source: Adapted from template provided by PRISMA: http://www.prisma-statement.org/.

We retrieved a total of 1,279 studies after the electronic database search, however after removing duplicates and screening the titles, abstracts and full texts as well as citation tracking and searching the reference lists of relevant articles, we arrived at nine articles that were eligible for inclusion in our review [36–44]. We initially added two excluded studies to the final included studies, however, during the data extraction, we discovered that these two studies did not meet the criteria for the comparison group. The first study used privately insured households as the comparison group instead of uninsured households [45]. In the second study, the entire sample consisted of households enrolled under the NHI and therefore, there were no uninsured households for comparison [46]. Consequently, we excluded both studies. The complete selection process thus yielded nine studies as shown in the PRISMA flowchart in Fig 1.

### Study characteristics

The nine eligible studies were published between 2011 and 2021 of which eight were conducted in Ghana and one in Nigeria. Also, eight studies were cross-sectional studies while one was a retrospective cohort study. From the included studies, five studies collected primary data by administering questionnaires to individuals or households while four studies analysed secondary data (Table 2). The sample size of the households ranged from 2,418 to 16,772 while that of the individuals ranged 196 to 11,617. Five of the studies had their study sample drawn from the general population irrespective of their health status while four studies drew their study sample from surgical patients on ward admission, tuberculosis patients, seriously injured children and employees in federal or state sector jobs (Table 2).

**Table 2:** Summary characteristics of included studies.

Publication details	Study Characteristics							
First author, year [citation]	Country/Region	Study objectives	Study design	Study period	Recall period	Sampling unit	Sample size	General health status
Flannery, 2020 [36]	Nigeria/Akwa Ibom state	To examine the relationship between enrolment in a NHI scheme and household health expenditures of federal and state workers in Akwa Ibom, Nigeria.	Cross-sectional survey	2018	4 weeks	Individual	538	Federal or state sector employees irrespective of health status.
Aryeetey, 2016 [37]	Ghana/ Central and Eastern region	To analyze the effect of health insurance on household OOP expenditure, CHE and poverty.	Cross- sectional survey	2009–2011	1 month	Household	3,128* 3,128¶	General population irrespective of health status
Fiestas Navarrete, 2019 [38]	Ghana (data from GLSS 6)	To evaluate the effect of health insurance on financial risk protection and utilization among high-risk and vulnerable beneficiaries with and without limited geographic accessibility to care	Cross-sectional survey	2012–2013	N/R	Household	16,744	General population irrespective of health status
Kusi, 2015 [39]	Ghana, Kwaebibirem/ Asutifi and Savelugu–Nanton	To examine the effect of the NHI scheme on OOP health expenditure and how it protects households against CHE.	Cross-sectional survey	02.2011–04.2011	4 weeks	Household	2,418	General population irrespective of health status
Nguyen, 2011 [40]	Ghana/ data from 2007 survey in Nkoranza and Offinso)	To evaluate the impact of the country’s NHI scheme on households’ OOP spending and CHE.	Cross-sectional survey	2010	12 months	Individuals	11,617	General population irrespective of health status
Okoroh, 2020 [41]	Ghana/Korle Bu Teaching Hospital (KBTH)	To test the hypothesis that insurance makes a difference in OOP expenditures for surgical care and CHE by comparing rates of financial catastrophe for insured versus uninsured surgical patients at a single institution, the KBTH.	Cross-sectional survey	01.02.2017–01.10.2017	3 months	Individual	196	Patients admitted on the general surgery ward
Pedrazzoli, 2021 [42]	Ghana (data of TB patients at public health facilities across Ghana)	To answer the following questions: 1) What are the key drivers of costs and catastrophic costs for TB-affected households? 2) If NHI scheme is a driver, what would be the expected changes in experienced costs of expanding the national health insurance scheme to all TB patients?	Cross-sectional survey	2016	N/R	Individual	690	TB patients
Stewart, 2021 [43]	Ghana/ Komfo Anokye Teaching Hospital (KATH)	To determine the effect of the NHI scheme on timeliness of care, outcomes, and household CHE of seriously injured children at a tertiary hospital in Ghana.	Retrospective cohort study	01.01.2015–31.12.2016	N/R	Individual	263	Seriously injured children
Kwakye, 2018 [44]	Ghana (data from GLSS 6)	The general objective of the study was to determine the effect of NHI scheme coverage on CHE among households in Ghana.	Cross-sectional survey	2012–2013	N/R	Household	16,772	General population irrespective of health status

**Note**:

* Data collected in the year 2009,

^**¶**^ Data collected in the year 2011, **N/R-**Not Reported

**NHI**: National Health Insurance. **CHE**: Catastrophic health expenditure. **OOP**: Out-of- pocket. **TB**: Tuberculosis.

**GLSS**: Ghana Living Standard Survey

In all nine studies the insurance type was the NHI, however the criteria for classifying a household as insured differed across studies except for two studies where no criteria were reported. Furthermore, aside one study that assessed financial risk protection in terms of both CHE and impoverishment, all remaining eight studies assessed financial risk protection only in terms of CHE. The assessment of CHE was heterogenous across all studies due to the use of different thresholds in the assessment. Characteristics of the included studies are summarized in Tables 2 and 3.

**Table 3:** Summary characteristics of included studies (*continuation*).

Publication details	Exposure Characteristics			Outcome Characteristics			
First author, year [citation]	Insurance type	Criteria for classifying household as insured	Total number of insured (% insured)	Measure of financial risk protection	Proportion of CHE [impoverishment]		Effect of NHIS on CHE [impoverishment]
					Insured	Uninsured	
Flannery, 2020 [36]	NHI scheme	N/R	265 (49.3%)	**CHE**–Health expenditure above threshold of 40% monthly non-food expenditure.	3.39%	80.22%	**+’**
Aryeetey, 2016 [37]	NHI scheme	N/R	986 (31.2%)* 1,176 (37.5%)¶	**CHE**–OOP expenditure equal to or exceeding a certain payment capacity threshold that is 40% of non-food consumption expenditure.	18.4% * 7.1% ¶	36.1% * 28.7% ¶	**+**
				**Impoverishment**–Health spending that pushes a household’s total expenditure below the mean monthly food expenditure (minimum subsistence need) of the study sample which was set at GH¢60.72 (US$ 43.4).	[2.9%] * [2.8%] ¶	[4.9%] * [3.3%] ¶	[**+**]
Fiestas Navarrete, 2019 [38]	NHI scheme	Household head enrolled into NHI scheme	5452 (32.56%)	**CHE**–OOP health payments that absorb >10% of household non-food consumption.	N/R	N/R	**+’**
Kusi, 2015 [39]	NHI scheme	Two criteria; 1. Fully insured if all the members were insured at the time of the survey. 2. Partially insured if at least one member was insured.	681 (28.2%) ƒ 620 (25.6%) §	**CHE**—Household’s total OOP health expenditure exceeding 40% of total non-food expenditure.	2.94% ƒ 3.71% §	4.03%	**+’**ƒ **+** §
Nguyen, 2011 [40]	NHI scheme	Any individual with NHI scheme at the time of survey	4899 (42.2%)	**CHE**—Health spending exceeding 10% of income per capita.	2.1%	1.2%	**0**’
Okoroh, 2020 [41]	NHI scheme	Surgical patient on admission insured under NHI scheme	127 (65%)	**CHE**–OOP expenditures exceeding: 1) 10% of annual total household expenditures.	1) 87%	1) 98%	N/R
				2) 20% of the individual’s income.	2) 63%	2) 83%	
				3) 40% of household’s non-food expenditures.	3) 58%	3) 94%	
Pedrazzoli, 2021 [42]	NHI scheme	TB patients insured under NHI scheme	562 (81%)	**CHE**–Household total TB-related costs (direct and indirect) exceeding 20% of their estimated pre- diagnosis annual household income	65%	59%	**0**’
Stewart, 2021 [43]	NHI scheme	Children insured by NHI scheme	184 (70%)	**CHE**–Health expenditure above threshold of 10% annual household income.	53.4%	84.8%	**+’**
Kwakye, 2018 [44]	NHI scheme	At least one member of household with NHI scheme coverage	11,829 (67.87%)	**CHE** 1) Household’s annual total OOP health payments (excluding hospitalization) equal or exceeding 10% of household’s non-food expenditure.	1) 5.74%	1) 7.21%	1) **+**.
				2) Household’s annual total OOP health payments (excluding hospitalization) equal or exceeding 40% of household’s non-food expenditure.	2) 0.35%	2) 0.31%	2) **0**

**Note**:

* Data collected in the year 2009,

^**¶**^ Data collected in the year 2011,

^**ƒ**^ Fully insured households,

^**§**^ Partially insured households, **N/R**: Not Reported

**Effect measures**: “**+**” Positive effect, “**–**” Negative effect, “**0**” no statistically significant effect (p-value >0.05)

**NHI**: National Health Insurance. **CHE**: Catastrophic health expenditure. **OOP**: Out-of- pocket. **TB**: Tuberculosis

### Quality appraisal

The quality of the included studies ranged from medium to high (Table 4). The common issues that affected the quality included no clear statement on the inclusion criteria (n = 2), no reliable measurement of the exposure (n = 2), no statement on identified confounding factors (n = 1), no statement on strategies to address confounding (n = 1) and no reliable measurement of outcome (n = 1). Also, the included cohort study did not have clear statements on the follow up time, the completeness of follow up as well as reasons for loss to follow up (Table 4). The overall probability of agreement between the two reviewers was judged as moderate (Cohen’s κ = 0.45) while the overall percentage agreement between the reviewers was 88% with the percentage agreement for each question ranging between 75% and 100% (Table 4).

**Table 4:** Quality appraisal using JBI checklist.

Checklist Items	Flannery 2020 [36]	Aryeetey 2016 [37]	Fiestas Navarrete, 2019 [38]	Kusi, 2015 [39]	Nguyen, 2011 [40]	Okoroh, 2020 [41]	Pedrazzoli.2021 [42]	Stewart, 2021 [43]*	Kwakye, 2018 [44]	% inter-reviewer agreement	Cohen’s kappa (κ) score
1. Clear inclusion criteria	No	Yes	Yes	Yes	Yes	Yes	Yes	**–**	No	75%	**–**
2. Detailed description of subjects and settings	Yes	Yes	Yes	Yes	Yes	Yes	Yes	Yes	Yes	100%	**–**
3. Valid and reliable measurement of exposure	No	No	Yes	Yes	Yes	Yes	Yes	**–**	Yes	75%	**–**
4. Objective, standard measurement criteria of condition	Yes	Yes	Yes	Yes	Yes	Yes	Yes	**–**	Yes	100%	**–**
5. Confounding factors identified	Yes	Yes	Yes	Yes	Yes	No	Yes	Yes	Yes	77.8%	**–**
6. Strategies to handle confounding stated	Yes	Yes	Yes	Yes	Yes	No	Yes	Yes	Yes	77.8%	**–**
7. Valid and reliable measurement of outcome	Yes	Yes	Yes	Yes	Yes	Yes	Yes	Yes	No	88.9%	**–**
8. Appropriate statistical analysis	Yes	Yes	Yes	Yes	Yes	Yes	Yes	Yes	Yes	100%	**–**
9. Exposed & unexposed from same population	**–**	**–**	**–**	**–**	**–**	**–**	**–**	Yes	**–**	100%	**–**
10. Similar measurement of exposed & unexposed	**–**	**–**	**–**	**–**	**–**	**–**	**–**	Yes	**–**	100%	**–**
11. All participants free of outcomes at start of study.	**–**	**–**	**–**	**–**	**–**	**–**	**–**	Yes	**–**	100%	**–**
12. Sufficient follow up time for outcomes to occur.	**–**	**–**	**–**	**–**	**–**	**–**	**–**	No	**–**	100%	**–**
13. Follow up completed/ reasons for loss to follow up reported.	**–**	**–**	**–**	**–**	**–**	**–**	**–**	No	**–**	100%	**–**
14. Use of strategies to address incomplete follow up.	**–**	**–**	**–**	**–**	**–**	**–**	**–**	No	**–**	100%	**–**
**Total Score**	6/8	7/8	8/8	8/8	8/8	6/8	8/8	8/11	6/8	88%	0.45
**OVERALL APPRAISAL**	**Moderate quality**	**High quality**	**High quality**	**High quality**	**High quality**	**Moderate quality**	**High quality**	**Moderate quality**	**Moderate quality**		**Moderate agreement**

**Note**:–Not Applicable.

* Retrospective Cohort study.

JBI checklist;

• **Cross-sectional studies**: High quality (7–8), moderate quality (5–6) and low quality (0–4)

• **Cohort studies**: High quality (10–11), moderate quality (7–9) and low quality (0–6)

Cohen’s kappa (κ) score interpretation;

• 0.01–0.20 slight agreement

• 0.21–0.40 fair agreement

• 0.41–0.60 moderate agreement

• 0.61–0.80 substantial agreement

• 0.81–1.00 almost perfect or perfect agreement

### Effect of NHI on CHE and impoverishment due health spending

The included studies reported different effects of NHI on CHE at different thresholds of household total income (expenditure) and non-food expenditure. In more than half of the included studies (5 out of 9), NHI was reported to have a positive effect (protective factor) on CHE [36–39, 43]. In addition, one of these five studies reported a positive effect of NHI on impoverishment due to health spending [37]. Furthermore, one out of the nine included studies reported both a positive effect and a no statistically significant effect of NHI on CHE at different thresholds of CHE [44]. In two out of the nine included articles, there was no statistically significant evidence that NHI had an effect on CHE [40, 42]. One out of the nine studies did not report on the effect of NHI on CHE [41]. No study reported a negative effect of NHI on CHE. The summary of the effect of NHI and impoverishment due to health spending is presented in Table 3.

### Proportion of insured households incurring CHE and impoverishment due to health spending

Eight studies assessed the proportion of households that incurred CHE among insured households [36, 37, 39–44]. In seven of these studies, the proportion of households encountering CHE was lower among insured households than uninsured households. In three out of these seven studies, more than 50% of insured households incurred CHE (ranging between 53.4% and 87%) [41–43]. The remaining one out of the eight studies reported a greater proportion of insured households incurring CHE as compared to uninsured households (Table 3) [42]. Only one study reported a proportion of insured households impoverished (below poverty line set at $43.4 per month) due to health spending as 2.9% and 2.8% from data collected in 2009 and 2011 respectively [37]. The summary of the proportion of households that encountered CHE and impoverishment is presented in Table 3.

### Determinants of CHE and impoverishment due to health spending

Six of the included studies reported on determinants of CHE [37, 39, 41–44] while one reported on the determinants of impoverishment due to health spending [37]. The identified determinants were related to the demographic and the health profile as well as the socio-economic circumstances of the households. A large household size, a high number of children under 5 years in a household, a female-headed household and a long distance between a household and a health facility were some demographic determinants of CHE. Furthermore, identified health related determinants of CHE included the health profile of households with at least one member having a chronic NCD, households with any child with severe injuries or any member undergoing surgical procedures or receiving in-patient care. In addition, households with members undergoing diagnostic tests not covered by the Tuberculosis (TB) Programme or NHI schemes such as chest radiography or tests for co-morbidities such as liver function test, undergoing treatment for multidrug resistant tuberculosis (MDR-TB) as well as those with members who make payments for medications were also identified as determinants of CHE. Further, the determinants of CHE related to socio-economic situation of households included patients from poor households, households with no or low level of education and households with unemployed individuals. Finally, the determinants of impoverishment due to health spending reported included large households, households with members who use in-patient services or those with low level of education as well as unemployed members.

### Effect of NHI on health outcomes and health service utilization

One study reported health outcomes as mortality in terms of number of insured and uninsured patients dying while on admission. In this study, three cases of mortality were reported out of the 184 insured patients while no case of mortality was reported out of the 79 uninsured patients. However, the association between NHI and patient mortality was not assessed in the study [43]. Health service utilization among households with insurance was reported by three studies but only one study reported a statistically significant positive effect between NHI and health service utilization and concluded that households with NHI were more likely to utilize health services compared to uninsured [38]. The two remaining studies reported an increased frequency of health service utilization among households with NHI as compared to uninsured households however no statistical analysis was performed to determine the measure of effect between NHI and health service utilization [36, 41].

### Financing mechanism of NHI systems in Ghana

As reported from the eight included studies conducted on Ghana [37–44], the NHI scheme in Ghana established in 2003 relies on diversified set of funding sources which includes a 2.5% levy on goods and services under the Value Added Tax (VAT) which contributes about 70% of the pooled funds. Additionally, 2.5% of the Social Security and National Insurance Trust (SSNIT) contributions are automatically deducted from salaries of formal sector workers and contributes about 20–25% of the pooled funds. The remaining 5% of funds come from voluntary income adjusted premiums by adults (18–69 years) in the informal sector.

However, portions of the Ghanaian population are exempted from payment of premium and they include pregnant women and their newborn, children under 18 years whose parents are enrolled under the NHI scheme, SSNIT pensioners, the elderly over 70 years, impoverished populations and individuals with mental disorders. Those exempted from premiums make up about 60% of the entire insured population enrolled under the NHI scheme and enjoy the full benefits that other premium paying members are entitled to.

Furthermore, based on the description from the included studies, the benefit package under the Ghana NHI scheme covers about 95% of the country’s health conditions and comprises of outpatient care including medications and laboratory diagnostics, in-patient services, surgical and obstetric care, treatment of cervical and breast cancers, basic oral health and ophthalmological services and emergency and trauma care. Despite the extensive conditions included in the benefit package, some services are still excluded under the NHI scheme and these services consist of other cancer treatment aside cervical and breast cancers, dialysis for chronic renal failure, antiretroviral medications and specialized care, medications and diagnostics for the management of trauma such as advanced diagnostic imaging, prosthetics, rehabilitation and mortuary fees.

Moreover, the included study reported that the NHI agency is the regulatory body of the NHI scheme in Ghana and is responsible for setting the premium and registration fees, pooling funds, negotiating benefit packages, accrediting and paying health service providers and ensuring quality service from health service providers. The administrative function of the NHI agency which includes the collation of insurance claims is decentralized to the district level. Finally, the service providers receive payment within four weeks of submission of their insurance claims [37–44].

### Financing mechanism of NHI systems in Nigeria

The study conducted on Nigeria reported that the NHI scheme was launched in 2005 and is made up of three packages with different mechanisms of financing. These packages include the Formal Sector Social Health Insurance Programme (FSSHIP), the Urban Self-Employed Social Health Insurance Programme (USSHIP) and the Rural Community Social Health Insurance Programme (RCSHIP).

This study focused on FSSHIP and as such described its financing mechanism [36]. It reported that the funding for the FSSHIP comes from an annual premium that is 15% of an employee’s annual basic salary which is a shared contribution between the employee (5%) and the employer (10%). Public and private sector workers, the armed forces, the police, para-military organizations and tertiary students are the main targets of the FSSHIP however this NHI scheme is open to voluntary contributors. No groups are exempted from this health insurance scheme.

Furthermore, the study mentioned that the benefit package under the FSSHIP in Nigeria includes preventative care (immunizations), out-patient services, in-patient care up to 15 days per year, obstetric care for up to four live births, eye and dental care, prostheses and prescribed medications and diagnostic tests available on the national recommended lists. Excluded services under the FSSHIP comprise of palliative care for terminal illnesses, antiretroviral medications, chronic diseases (diabetes and hypertension), renal dialysis and spectacle and contact lens provision.

It was also reported that the Health Maintenance Organizations (HMOs) are the main organizations responsible for collecting premiums, pooling funds, paying for health services as well as ensuring quality of care by health service providers under the FSSHIP. The HMOs are usually publicly or privately owned limited liability companies who aim at providing cost-effective healthcare delivery. Payment of providers under the FSSHIP depends on whether care received by insured is at primary healthcare facility, secondary or tertiary healthcare facility: for primary care payment is by capitation whereas for secondary and tertiary care, payment for services provided is based on fee-for-service [36].

### Summary of limitations of included studies

Limitations reported in the final included studies were related to the study design, information recall, outcomes assessment, identification of confounders and generalization of findings to the entire population of countries. The cross-sectional nature of most of the study designs gathered data at one point in time and made it impossible to follow up on households to ascertain if they incurred recurrent CHE. Furthermore, the retrospective cohort study had similar limitations in that, it did not follow children longitudinal even though seriously injured children may require numerous health visits within the course of their treatment which will be associated with OOP health expenditure which predispose to CHE.

Studies also observed that it was difficult for certain households where members were informal sector workers or had unstable or seasonal income to accurately recall their incomes. One study reported a short recall period as a limitation because it may underestimate health services utilization and subsequent OOP payments which may be associated with CHE. Some studies used the national median household expenditure to estimate CHE which may not reflect the median annual household expenditure of the study sample and give imprecise results.

Another limitation mentioned was that studies did not explore if households had to borrow or sell assets to make payment for health services and how they coped with CHE and its impoverishing effects. Possible confounding factors such as type of illness and disabilities among household members, existing healthcare systems and availability of social protection policies were also not identified in certain studies though these may influence the effect of NHI on CHE. The results of some studies were also restricted to subgroups of the population such as formal sector employees, patients on admissions and certain districts. This limited the generalizability of the results to the entire population of the various countries. Lastly, one study reported a selection bias towards insurance with insurance coverage among study participants two times more than that of the national average as such limiting the extrapolation of the findings to the national level.

### Summary of recommendations by included studies

The included studies recommend numerous strategies to develop NHI schemes to protect households against CHE and impoverishment due to health spending. Most studies advocated the need for future research to better explore factors that allow for an effective and well-functioning NHI scheme. Studies recommended impact evaluations using other study designs to clearly identify the factors of NHI schemes that positively impact OOP payments. One study mentioned the importance of conducting benefit incidence analysis to document how different households with different wealth status benefit from NHI schemes. In-depth case studies were also recommended to explore how NHI schemes are operationalized and to assess the quality of care received by households enrolled under NHI. Advocacy was made for more qualitative studies to explore the reasons for low NHI enrollment rates among rural dwellers to help extend coverage to include these populations.

Additionally, gathering of evidence to identify the exact OOP health expenditure which predisposes households with NHI to CHE was recommended by some studies. In relation to TB management under NHI schemes, it was recommended that further investigations be made into why the current NHI does not effectively protect against CHE among insured TB patients. Other studies recommended development towards a more inclusive and equitable NHI scheme that will ensure that populations that are vulnerable due to their socio-economic and geographic characteristics will also benefit from the financial risk protection as well as the health services utilization effect of NHI schemes.

Some studies recommended that physical access to health facilities should be improved by considering transportation cost as part of the benefit package of NHI schemes. It was also recommended that measures be put in place to ensure strict adherence of health facilities to benefit packages under NHI schemes. Also, a study reported that the rates of payment of health facilities by insurance agencies for surgical care under the NHI scheme should be improved. Finally, to better understand the overall impact of NHI on CHE, it was recommended that studies with reliable nationwide datasets be conducted to get results that can be extrapolated to the general population to guide policymakers in decision making for the effective implementation and operationalization of NHI schemes nationwide.

## Discussion

At the time of this review, this appeared to be first systematic review to ascertain the extent of financial risk protection among households enrolled under NHI schemes in the entire West Africa as well as to summarize potential learnings and recommendations for countries in the subregion. The majority of the included studies found enrollment into the NHI to be a protective factor against CHE, however, these same studies reported some households enrolled under NHI schemes continue to incur CHE and impoverishment due to health spending.

A systematic review on the effect of NHI on financial risk protection conducted in 2019 among LMICs reported a protective effect of NHI on CHE in majority (9 out of 14) of the included studies [47]. This finding is consistent with our review and suggests that indeed households enrolled into NHI schemes are more likely to have reduced or no OOP health expenditure and thus overall, least likely to incur CHE or be impoverished as compared to uninsured households. However, from our review, the protective effect of NHI on CHE varied according to thresholds of CHE (see Table 3). Overall, there are inconsistencies in the thresholds of CHE at which a positive effect of NHI can make a significant difference among insured households in LMICs. This may be because depending on the socio-economic status of a household, small amounts of health expenditure will be catastrophic for a household if the set threshold of CHE is low. For instance, a poor household will have an increased risk of encountering CHE after making OOP payments if a threshold of 10% is used while this same household will not encounter CHE at a threshold of 40% [48, 49].

For the proportion of insured households incurring CHE and impoverishment due health spending, a household survey conducted in Iran in 2015 observed similar findings as reported in our review (see Table 3) that insured households continued to incur CHE [50]. Just like Ghana and Nigeria, Iran is a LMIC and insured households in these countries may be subjected to high OOP payments which will predispose to CHE [51, 52]. Furthermore, in LMICs cost of indirect payments such as cost of transportation to health facility (especially in cases of emergency) as well as feeding while on admission may not be accounted for in benefit package of NHI schemes and as such may result in CHE among insured households [47]. The proportion insured households incurring CHE was found to be more than 50% in three of our included studies (see Table 3). These findings were higher compared to the recent WHO Global Monitoring Report on Financial Protection in Health that reported that in 2017, in the African region, 8.4% and 2.0% of households incurred CHE at the 10% and 25% thresholds respectively [53]. This difference could be due to the fact that, the three studies included patients on admission at the general surgery ward, TB patients and seriously injured children respectively. Thus, suggesting a high degree of OOP payments among these patient populations despite their conditions being included in the benefit package of the current NHI schemes based on our included studies [36–44]. Overall, the increased proportion of insured households incurring CHE in parts of West Africa suggests a possible weakness of the NHI in its current state in guaranteeing financial risk protection of the insured household and this may be a significant barrier to achieving UHC in West Africa.

The WHO Global Monitoring Report on Financial Protection in Health stated that in 2017, the proportion of households in the African region falling below the internationally standardized poverty line of $3.20 per day due to health expending was 1.1% [53]. However, in one included study, we observed that almost 3% of insured households made health expenditure that pushed them below the poverty line set at $43.4 per month in 2009 and 2011 [37]. The difference in the set poverty lines could be linked to possible externalities such as inflation as well as local or regional differences or methodologies. Researchers should therefore endeavor to employ standardized context-specific poverty lines while conducting research in this field to facilitate a better comparison of data on impoverishment due to health spending.

### Financing mechanism of NHI systems in Ghana and Nigeria

From this review, we learned that the voluntary purchase of insurance premiums by informal sector workers in Ghana as well as the pooling of funds at subgroup level according to shared characteristics of the population instead of a nationwide level pooling in Nigeria are ineffective mechanisms of mobilizing sufficient funds to finance the NHI schemes. Subsequently, both countries are plagued with low availability of funds for payment of health services. Additionally, the large percentage of the insured population who are exempted from paying premiums (approximately 60%) places a burden on those who pay premiums and this results in not only insufficient but also inequitable pooling of funds. The aforementioned West African NHI systems differ from the German SHI system where payment of premiums is compulsory for a large part (90%) of the population and funds are equitably pooled based on the concept of solidarity where the rich and healthy subsidize for the poor and sick [54, 55]. Despite the fact that the German SHI system is from a developed country, it can be used to make comparisons because as one of the oldest and well-established health insurance systems, it has formed the basis for many health insurance systems across the world [12]. Indeed, one of the major successes of the German SHI is evidenced by its effective pooling of funds nationwide [54, 55].

All included studies mentioned a wide range of services included in the benefit package of the NHI schemes which is highly desirable since it is in line with the drive towards achieving UHC. However, it was reported that certain chronic NCDs are not included in the benefit packages of NHI schemes in Ghana and Nigeria. In a study conducted across LMICs in 2020, it is evidenced that the prevalence of CHE and impoverishment due to health spending is highest among households with NCDs [56]. Furthermore, a study conducted among 48 LMICs in 2015 confirmed that health insurance reduces treatment inequities and socioeconomic disparities associated with NCDs [57]. Considering that the burden of NCDs across the African continent is increasing [58, 59], the identified gap in the NHI scheme contributes to and exacerbates the inequities of NCDs management. Thus, this greatly undermines the financial risk protection effect of NHI which can be a major hinderance to the drive towards UHC. Countries should therefore consider accounting for prevalent context-specific NCDs and their complications when developing the benefit packages of their NHI systems.

The NHI agency which regulates the NHI scheme in Ghana decentralizes its administrative function to the district level. Evidence from Pakistan suggests that decentralization of the healthcare sector to subnational levels encourages the growth of government measures to improve health service delivery [60]. Therefore, it can also be considered that in Ghana, the decentralization of the administrative functions can contribute to improving the implementation and operationalization of NHI schemes by facilitating the collation and vetting of insurance claims from health facilities, promoting accountability of subnational health services as well as encouraging enrollment into NHI schemes. However, a major disadvantage of decentralization is that high costs of human resources and infrastructure that arise from having various district offices across the country places a financial burden on the public funds of the country. Thus, to ensure the sustainability of NHI schemes, it is empirical for governments to consider innovative ways to fund a decentralized administrative system. In addition, employing digital technology systems in a decentralized organization will play a significant role in simplifying the administrative procedures.

The reimbursement of health facilities for services provided is a vital component of NHI schemes but the included studies from Ghana did not give much information on this. The study from Nigeria reported that the mode of payment for health services depends on which health facility level patients seek care from. This consists of capitation payment at primary care level and fee-for-service at the secondary and tertiary levels. These payment methods differ from that of Germany where payment for ambulatory services is based on fee-for-service [61] and that of hospital services is based on aG-DRG (Diagnostic-related groups) where diagnoses requiring similar treatments are classified into groups and the number of healthcare workers required to manage these conditions are also accounted for before reimbursements are made to the hospitals [62]. The inconsistencies in the method of reimbursement of health facilities between both countries could be due to the existing health policies and health systems organization as well as payment negotiations among other factors. Moreover, the capitation has been found to increase the tendency for healthcare facilities to provide poor quality care while fee-for-service has been linked to escalation of costs [63]. Evidence suggests that the aG-DRG mode of reimbursement for hospitals which as of 2022 is the new DRG system for Germany plays a key role in promoting quality care and ensuring equity in the payment for health services [62, 64, 65]. This new aG-DRG system came into the forefront of health financing discussions in 2021 during the Corona Pandemic which exposed the weakness in the previous DRG system that did not take into account the workforce required to manage patients [66]. Hence, for the improvement of reimbursement of health facilities under NHI schemes in Nigeria and West Africa as a whole, not only should past experiences or systems be considered but more importantly, current trends as well as cultural preferences, perspectives and views of health professionals should be taken into account so as to develop a robust and sustainable NHI system.

### Recommendations

Based on the findings of this systematic review, the following recommendations on future research focus and policy strategies to improve NHI schemes will be vital in ensuring financial risk protection to facilitate the drive towards UHC in West Africa. Since majority of West African countries are French speaking countries, future systematic reviews should include French as an inclusion criterion to capture relevant articles published in French. This will allow for a more comprehensive exploration of the financial risk protection effect of NHI schemes in West Africa. Furthermore, nationwide surveys should be carried out to explore the extent to which existing NHI schemes protect against CHE and impoverishment due to health spending, this will allow for easy generalizability of the results and enable policymakers to make decisions and implement interventions that include the entire population. It is also recommended that researchers have a common criterion for describing household as insured as well as having a common context-relevant threshold for measuring CHE. These common measures will allow for easy comparison and analysis of results to draw relevant conclusions. Future research on financial risk should also consider measuring impoverishment due to health spending in combination with CHE because governments and policymakers are likely to be more sensitive to poverty measures and react accordingly. Continuous studies should also be conducted on the benefit package of NHI schemes with respect to the prevailing disease conditions associated with CHE and impoverishment due to health spending in countries. This may help introduce benefit packages that are not only context-relevant but also effectively protect against CHE and impoverishment due to health spending.

In relation to the policy recommendations, premium contributions should be compulsory for the entire population of countries for NHI schemes to effectively pool sufficient funds for health services. Furthermore, exemptions of payment of premiums should be limited to impoverished populations who would be identified through a robust registration process for these vulnerable groups. In the drive towards financial risk protection and UHC, social protection policies should be rolled out to improve the socio-economic circumstances of the citizens of countries. Also, administrative processes such as vetting of claims should be digitized and decentralized to facilitate effective collation and efficient analysis. Additionally, to reduce the risk of moral hazard, strategies such as well-designed payment of deductibles and coinsurance are recommended. Capacity building and sufficient resource allocation to the field of research focused on financial risk protection and UHC is recommended to equip policymakers with the evidence needed for decision making. Finally, West Africa is under one economic community (ECOWAS) and has a regional health organization known as the West African Health Organization (WAHO) that is responsible for ensuring and promoting the health of citizens and fostering regional governance in health through the investment in health systems research in West Africa [67]. Due its functions, WAHO is in a desirable position to work together with ECOWAS member states to co-design a robust context-specific NHI scheme that could be adopted and implemented by member states. These recommendations complement the aforementioned recommendations summarized from the included studies (see Results) and will overall contribute to improving the financial risk protection effect of NHI schemes and facilitate the drive towards UHC.

### Limitations of the study

Despite the robust methodology of this review, several limitations have to be mentioned. First of all, the findings are based on only nine studies with majority of the studies from Ghana and as such these findings cannot be generalized to all countries in West Africa. Also, the exclusion of literature published in French was a major limitation to this review. Furthermore, only two of the nine included studies had nationally representative data which also limits the generalization of the overall findings. Moreover, because of the timeline set for this study, articles published after 1st May 2022 were not included and as such results presented in this systematic review may not reflect recent developments after this date. Additionally, since only one study reported on impoverishment due to health spending, conclusions drawn on this measure of financial risk protection cannot be generalized to the entire West Africa. Another limitation was the high degree of heterogeneity among included studies due to difference in the criteria for classifying households as insured as well as the thresholds for accessing CHE. Finally, the nine included studies encountered several limitations which may be relevant for future studies and are summarized under the Results section.

## Conclusion

This systematic review provides evidence that households insured under NHI schemes in some West African countries (Ghana and Nigeria) are more likely to be protected against CHE and impoverishment due to health spending compared to uninsured households as reported in six out of the nine included studies. However, households insured under NHI schemes continue to incur CHE and impoverishment due to health expenditure with three out nine studies reporting more than 50% of insured households incurring CHE. Reasons for this are numerous and interlinked and can be related to households’ demographic and socio-economic characteristics as well as their health profiles. The most relevant determinants of CHE and impoverishment due to health spending identified among households were poverty and unemployment, presence of chronic diseases and indirect OOP health expenditure such as transportation costs to health facilities. To protect households in West African countries against the devastating effects of financial loss due to health spending, further research using nationally representative data needs to be conducted to ascertain the extent of NHI schemes on financial risk protection in West African countries in order to have relevant data to guide evidence-based decision making. Governments should also consider focusing on rolling out compulsory nationwide NHI premium contributions while implementing effective social protection schemes to reduce the incidence of CHE and impoverishment due to health spending. UHC in West Africa can be made attainable and the role of a multinational collaboration between West African countries to co-design a sustainable context-specific NHI system based on solidarity and equity will go a long way in advancing the drive towards UHC.

## Supporting information

S1 Table Search strategies. This is a description of our comprehensive list of search strings used to identify all studies in the various electronic databases.
(PDF)

S1 Data Complete data extraction form. This contains the complete data extracted from included articles in this review.
(XLSX)

S1 Checklist PRISMA 2020 checklist. (PDF)
