# Supplementary material for: Do national health insurance schemes guarantee financial risk protection in the drive towards Universal Health Coverage in West Africa? A systematic review of observational studies
Source: PLOS Glob Public Health. 2023 Aug 9;3(8):e0001286. doi: 10.1371/journal.pgph.0001286 (PMC10411819; doi:10.1371/journal.pgph.0001286)
Supplement: S1 Table — This is a description of our comprehensive list of search strings used to identify all studies in the various electronic databases. (PDF) [file pgph.0001286.s001.pdf]

SI1 Table: Search Strategies

Database	Search number	Search String	Articles found
PubMed/Medline	#1	“Insurance, Health”[Mesh] OR “Universal Health Insurance”[Mesh] OR “National Health Programs”[Mesh] OR “health insurance*”[tw]	275,513
	#2	“Africa, Western”[Mesh] OR “west* Africa*”[tw] OR Benin[tw] OR “Burkina Faso”[tw] OR “Cape Verde”[tw] OR “Cabo Verde“[tw] OR “Cote d’Ivoire”[tw] OR “Ivory Coast”[tw] OR Gambia[tw] OR Ghana[tw] OR Guinea[tw] OR “Guinea-Bissau”[tw] OR Liberia[tw] OR Mali[tw] OR Niger[tw] OR Nigeria[tw] OR Senegal[tw] OR “Sierra Leone”[tw] OR Togo[tw]	273,215
	#3	“Risk Sharing, Financial”[Mesh] OR “Poverty”[Mesh] OR “financial risk”[tw] OR “catastrophic health expenditure*”[tw] OR “catastrophic loss*”[tw] OR “financial loss*”[tw] OR impoverish*[tw] OR poverty*[tw]	75,397
	#4	“Universal Health Care”[Mesh] OR UHC[tw] OR "universal health"[tw] OR "health for all*"[tw]	10,604
	#5	#1 AND #2 AND (#3 OR #4), from 2005 - 2022	**327**
Web of Science	#1	ALL=("Health Insurance*")	51,240
	#2	(ALL=("west* Africa*") OR ALL=(Benin) OR ALL=("Burkina Faso") OR ALL=(“Cape Verde”) OR ALL=(“Cabo Verde“) OR ALL=(“Cote d'Ivoire”) OR ALL=(“Ivory Coast”) OR ALL=(Gambia) OR ALL=(Ghana) OR ALL=(Guinea) OR ALL=(“Guinea-Bissau”) OR ALL=(Liberia) OR ALL=(Mali) OR ALL=(Niger) OR ALL=(Nigeria) OR ALL=(Senegal) OR ALL=(“Sierra Leone”) OR ALL=(Togo))	451,349
	#3	(ALL=("Financial risk") OR ALL=("catastrophic health expenditure*") OR ALL=("catastrophic loss*") OR ALL=("financial loss*") OR ALL=(impoverish*) OR ALL=(poverty))	116,671
	#4	(ALL=(“Universal health") OR ALL=(UHC) OR ALL=("health for all"))	10,644
	#5	#1 AND #2 AND (#3 OR #4), from 2005 - 2022	**293**

Database	Search number	Search String	Articles found
CINAHL via EBSCOhost	#1	"Health Insurance*"	400,602
	#2	"west* Africa*" OR Benin OR "Burkina Faso" OR “Cape Verde OR “Cabo Verde” OR “Cote d'Ivoire” OR “Ivory Coast” OR Gambia OR Ghana OR Guinea OR “Guinea-Bissau” OR Liberia OR Mali OR Niger OR Nigeria OR Senegal OR “Sierra Leone” OR Togo	146,028
	#3	"Financial risk” OR "catastrophic health expenditure*" OR "catastrophic loss*" OR "financial loss*" OR impoverish* OR poverty	640,030
	#4	“Universal health" OR UHC OR "health for all"	51,896
	#5	#1 AND #2 AND (#3 OR #4), from 2005 - 2022	**262**
Embase via Ovid	#1	health insurance/ or health insurance*.mp.	181,001
	#2	(West* Africa* or Benin or Burkina Faso or Cape Verde or Cabo Verde or Cote d'Ivoire or Ivory Coast or Gambia or Ghana or Guinea or Guinea-Bissau or Liberia or Mali or Niger or Nigeria or Senegal or Sierra Leone or Togo).mp.	33,111
	#3	(Financial risk or catastrophic health expenditure or catastrophic loss or financial loss).mp. or poverty/ or poverty.mp. or impoverish*.mp.	74,354
	#4	universal health care/ or universal health.mp. or UHC.mp. or health for all.mp.	19,670
	#5	#1 AND #2 AND (#3 OR #4), from 2005 - 2022	**385**
Google scholar	#1	"Health insurance" AND "West Africa" AND "financial risk" OR "catastrophic health expenditure" OR "catastrophic loss" OR "financial loss" OR impoverish OR "universal health"	5,184
	Articles were ranked automatically based on relevance after which the first 500 articles were manually screened to find articles to be included at the titles and abstract screening stage.		**12**
